# A nomogram integrating ultrasound features of the primary tumor and axillary lymph nodes with clinicopathological factors to predict high axillary lymph node burden in invasive breast cancer

**DOI:** 10.3389/fonc.2026.1906231

**Published:** 2026-10-13

**Authors:** Xiuwei Li, Haocheng Qin, Yun’an Chen, Jiale Zhu, Ming Yu

**Affiliations:** The Affiliated Lianyungang Hospital of Xuzhou Medical University/The First People’s Hospital of Lianyungang, Lianyungang, Jiangsu, China

**Keywords:** axillary lymph node metastasis burden, invasive breast cancer, nomogram, primary lesion, ultrasound features

## Abstract

**Objective:**

To develop and validate a nomogram that integrates ultrasound features of the primary tumor and axillary lymph nodes with lymphovascular invasion (LVI) for early postoperative risk stratification of high axillary nodal burden (HNB; ≥3 positive nodes) in patients with node-positive invasive breast cancer.

**Methods:**

A total of 175 consecutive patients with node-positive invasive breast cancer who underwent surgery between July 2023, and October 2025 were retrospectively included in the development cohort, and an additional 86 patients treated during the same period comprised of the internal validation cohort. Two senior radiologists independently reviewed preoperative ultrasound images and clinicopathological data, including LVI assessed in postoperative surgical specimens. Univariate and multivariate logistic regression analyses were performed to identify independent predictors and construct the nomogram.

**Results:**

The proportions of patients with posterior echo attenuation, microcalcification, claw sign, angular margin sign, spiculation sign (significant long spicules/short spicules), suspicious axillary lymph node status, and lymphovascular invasion were all higher in the high-load group than in the low-load group (all *P* < 0.05). Multivariate regression analysis showed that the absence of the marginal angular margin sign (*OR* = 0.190, 95% *CI*: 0.084 - 0.432, *P* < 0.001) and the normal axillary lymph node status (*OR* = 0.208, 95% *CI*: 0.089 - 0.456, *P* < 0.001) were independent protective factors for HNB, whereas lymphovascular invasion (presence) (*OR* = 2.707, 95% *CI*: 1.363 - 5.377, *P* = 0.004) was an independent risk factor. ROC analysis showed that the combined detection of marginal angular margin sign, lymph node status, and lymphovascular invasion had a higher AUC value for predicting HNB than individual detections (*Z* = 4.176, 3.781, and 3.672, respectively; *P* < 0.05).

**Conclusion:**

The nomogram incorporating the angular margin sign, axillary lymph node ultrasound status, and LVI provides effective early postoperative risk stratification for HNB in patients with node-positive invasive breast cancer and may facilitate timely adjuvant treatment decision-making.

## 1 Introduction

Breast cancer is the most common malignant tumor in women worldwide, with invasive breast cancer accounting for over 80% of cases. It is one of the leading causes of cancer-related deaths among women (1). Unlike other pathological subtypes, invasive breast cancer has biological characteristics such as invasive growth, early occurrence of lymphatic vessel invasion, and regional lymph node metastasis. The status of axillary lymph node metastasis and metastasis burden of this type are the most important prognostic indicators, directly determining the clinical stage of patients, the surgical approach, and the formulation of adjuvant treatment strategies, and having a decisive impact on the long-term survival and quality of life of patients (2, 3). In recent years, landmark clinical studies, such as ACOSOG Z0011 and AMAROS, have completely transformed the traditional model of axillary management in breast cancer. Studies have confirmed that for patients with cT1–2 stage, clinically negative axillary lymph nodes, and a sentinel lymph node biopsy (SLNB) indicating a transfer burden of ≤ 2 lymph nodes, there is no significant difference in the long-term survival rate between simple SLNB and axillary lymph node dissection (ALND). However, it can significantly reduce the incidence of postoperative lymphedema, upper limb dysfunction, sensory abnormalities, and other complications and improve the quality of life of patients. For patients with a high load of axillary lymph node metastasis (≥ 3 lymph nodes), axillary lymph node dissection or intensified systemic treatment is still required to control the risk of local recurrence (4, 5). Therefore, accurately differentiating low- and high-load axillary metastases in patients with invasive breast cancer before surgery is crucial for achieving axillary management and avoiding excessive or insufficient treatment. Currently, the methods for preoperative assessment of axillary lymph node status mainly include imaging examinations such as ultrasound, breast MRI, ^18^F-FDG PET/CT, and preoperative biopsy. Ultrasound has become the preferred imaging method for preoperative axillary assessment in breast cancer owing to its advantages such as non-invasiveness, convenience, economy, no radiation, and good repeatability,. However, the sensitivity of conventional ultrasound in diagnosing axillary lymph node metastasis is only 60% to 70%, and it is greatly influenced by the operator’s experience. Its ability to identify micrometastases and low-load metastases is limited (6, 7). The SLNB is the gold standard for evaluating the status of axillary lymph nodes. However, as an invasive procedure, it still has a false-negative rate of approximately 5% to 10%, and it cannot provide a complete basis for surgical plan formulation before the operation. Breast MRI and ^18^F-FDG PET/CT have excellent diagnostic efficacy, but their high examination costs and low equipment popularity make them difficult to use as routine preoperative screening methods for early breast cancer (8–10). Preoperative biopsy pathological parameters can reflect the biological behavior of the tumor, but they cannot independently predict the preoperative load of axillary metastasis. However, there are obvious limitations to its clinical application. The reliability of the nomogram model in the context of precision diagnosis and treatment of tumors has been supported by multiple studies (11, 12). Several studies have constructed nomogram models to predict axillary lymph node metastasis in breast cancer. However, most of these models include a large number of indicators or contain complex radiomics features, making their clinical application challenging. Moreover, there are relatively few prediction models specifically targeting high axillary lymph node burden (High Nodal Burden, HNB) (≥ 3 metastatic lymph nodes), and the performance of these models still needs to be further improved.

Based on this rationale, we retrospectively analyzed the preoperative ultrasound features and clinicopathological data of 175 patients with node-positive invasive breast cancer to identify the independent predictors of HNB. We aimed to develop a simple and practical nomogram that integrates ultrasound features with LVI, which was assessed in postoperative surgical specimens, to facilitate early postoperative risk stratification for HNB. The model is intended to support timely adjuvant treatment decisions once pathological results become available, including the need for completion axillary lymph node dissection (if not already performed), the extent of radiotherapy fields, and the intensity of systemic therapy.

## 2 Data and methods

### 2.1 Research objects

Patients diagnosed with histologically invasive breast cancer between July 2023 and October 2025 at Lianyungang Hospital Affiliated to Xuzhou Medical University, who underwent primary breast resection and surgical intervention of axillary lymph nodes (including biopsy or dissection) were retrospectively collected. Eligibility for inclusion was contingent upon: (1) female patients aged 18–80 years; (2) postoperative pathology confirmed as unilateral single-focal primary invasive breast cancer; (3) breast and axillary ultrasound examinations were completed within 2 weeks before the surgery, and the ultrasound images and reports were complete; (4) surgical intervention for the primary breast lesion and axillary lymph nodes (SLNB and/or ALND); and (5) postoperative pathology confirmed the presence of axillary lymph node metastasis. The exclusion criteria were as follows: (1) multifocal or bilateral breast cancer; (2) received neoadjuvant chemotherapy, radiotherapy, or endocrine therapy before surgery; (3) pregnant or lactating women; (4) a history of other malignant tumors; (5) poor quality of ultrasound images, making it impossible to accurately interpret the signs; and (6) incomplete clinical and pathological data.

Based on the above inclusion and exclusion criteria, 175 patients were selected and divided into low- (84) and high-load (91) groups. The research protocol was approved by the Medical Ethics Committee of Lianyungang Hospital Affiliated to Xuzhou Medical University (Approval Number: KY-20260401003-01), and it was determined to comply with the relevant regulations for waiving written informed consent.

According to the predetermined inclusion and exclusion criteria, this study selected the final cohort from the initial 273 patients with invasive breast cancer who underwent surgery. The specific screening process is shown in **Figure 1**.

**Figure 1:**
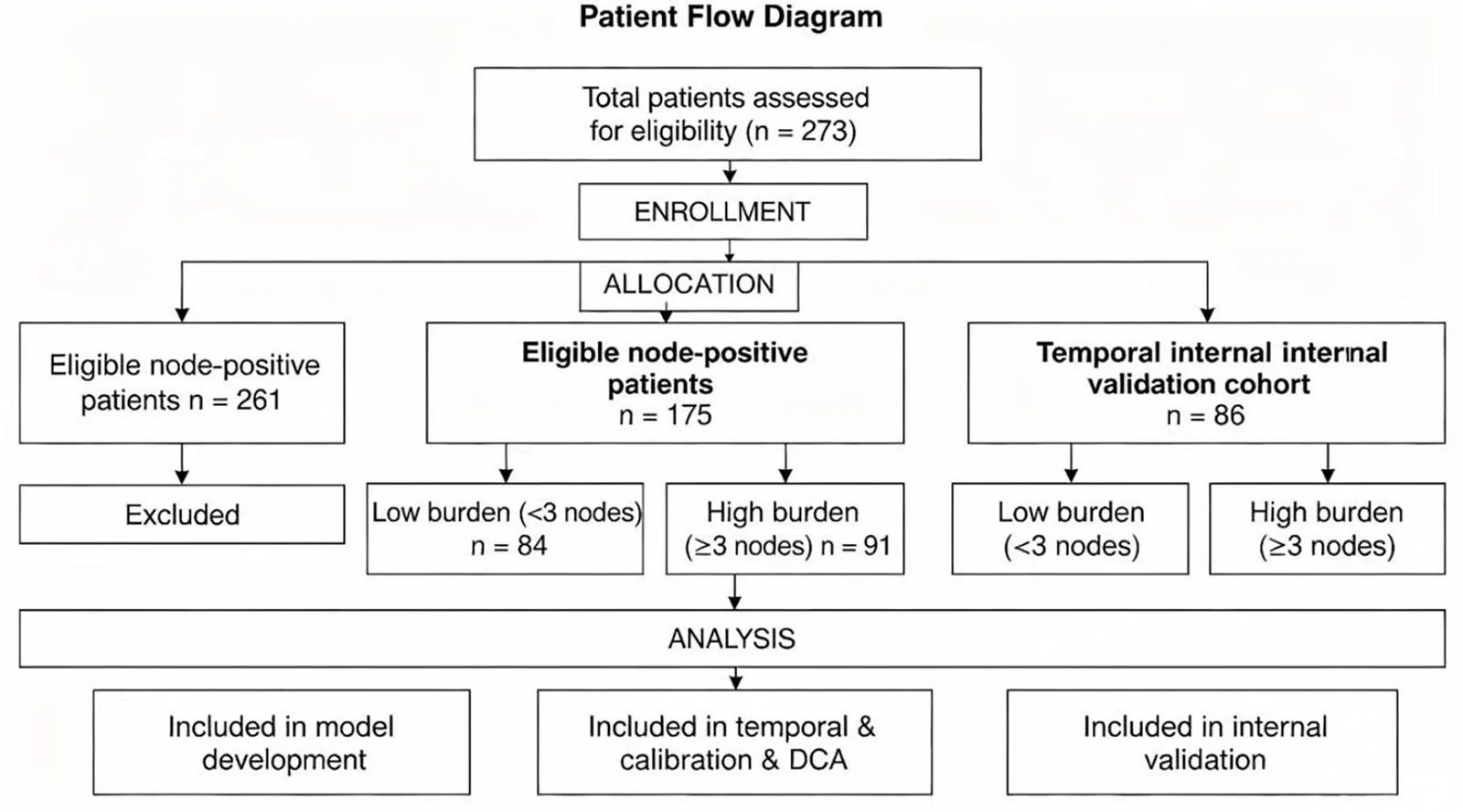
Patient flow diagram.

Patients were assigned chronologically to the model development and temporal validation cohorts according to their surgery date. Patients who underwent surgery between July 2023 and December 2024 (n = 175) comprised the development cohort, whereas those who underwent surgery between January and October 2025 (n = 86) comprised the temporal internal validation cohort. The validation cohort was not used for model development but to assess the model’s temporal generalizability. Patients with incomplete clinical, pathological, or ultrasound data were excluded; therefore, a complete case analysis was performed.

This flowchart illustrates the patient selection process, from the initial identification of 273 patients with invasive breast cancer to the inclusion of 175 patients in the model development cohort and 86 patients in the temporal validation cohort.

### 2.2 Acquisition of ultrasonic features

All patients underwent preoperative breast and axillary examinations using the Philips EPIQ 7 color Doppler ultrasound diagnostic instrument, with probe frequencies ranging from 5 to 12 MHz. The patients were placed in the supine position, with both arms raised to fully expose the bilateral breasts and axillae. Continuous scanning was performed on each quadrant of the breast, and the location, size, shape, margin, internal echo, posterior echo, calcification status, and blood flow signals of the lesion were recorded in detail. The bilateral axillae were routinely scanned to observe the size, shape, cortical thickness, lymph node structure, and blood supply pattern of the lymph nodes. All ultrasound images were stored in the hospital’s PACS system in DICOM format.

Two physicians with over 8 years of experience in specialized breast ultrasound diagnosis independently reviewed the stored ultrasound images, blinded to the postoperative pathological results and clinical data of the patients. The following ultrasound features of the primary lesion were recorded: the maximum diameter of the tumor (cm), aspect ratio (<1/≥1), posterior echo (unchanged/enhanced/decayed), type of calcification (none/minimal calcification/coarse calcification/clumped calcification), shape (regular/irregular/irregular), margin (clear/relatively clear/unclear/unclear), echo type (hyperechoic/mixed echo), echo uniformity (uniform/uniformity), angular margin sign of the margin (none/yes), claw sign (none/yes), spiculation sign (no spiculation/short spiculation/obvious long spiculation), Adler blood flow grade (0/1/2/3 levels), vascular morphology (no blood flow/dot-like/linear/branch-like/intrusion), and the quadrant where the lesion was located (outer upper quadrant/other).

Criteria for determining axillary lymph node status: Lymph nodes were considered suspicious for metastasis if any of the following features were present: (1) round shape or long-to-short-axis ratio (L/S) <2; (2) irregular or eccentric cortical thickening measuring ≥3 mm; (3) narrowing, displacement, or absence of the lymph node hilum; (4) microcalcifications within the lymph node; or (5) a nonhilar vascular pattern on color Doppler ultrasound (13).

The consistency of ultrasound sign interpretation by the two physicians was evaluated using Cohen’s Kappa coefficient. The results showed that the Kappa value for the angular edge sign was 0.89 (95% *CI*: 0.82 - 0.96), and the Kappa value for the status of axillary lymph nodes was 0.92 (95% *CI*: 0.86 - 0.98), indicating excellent consistency among the observers. If there was a disagreement in the interpretation of a certain sign by the two physicians, they would reach a consensus after jointly reviewing the images and having a thorough discussion.

Preoperative ultrasound examinations were performed by sonographers with at least 5 years of experience who were not involved in subsequent image interpretation. The stored ultrasound images were independently reviewed by two breast radiologists with more than 8 years of experience, who were blinded to the patients’ clinical and pathological data.

### 2.3 Clinical pathological indicators

Clinical pathological data of the patients were collected by searching the hospital electronic medical record system and the digital pathological report archiving system: age, histological grade (I, II, III) and lymphovascular invasion status (present/absent), hormone receptor expression (ER/PR), HER2 protein overexpression/gene amplification status, Ki-67 percentage reflecting tumor proliferation activity, and the number of axillary lymph node metastases confirmed by pathology.

The grouping of axillary lymph node metastasis burden was based on the results of the ACOSOG Z0011 trial. Lymph node metastasis of ≥ 3 was classified as a severe load, and < 3 as a mild load. According to the expert consensus of the 2013 St Gallen International Conference on Breast Cancer (14), receptor status was defined as follows: ① estrogen receptor and progesterone receptor expression was considered positive when ≥1% of tumor cells showed positive staining and negative when <1% showed positive staining; ② human epidermal growth factor receptor 2 (HER2) status was classified as positive for an immunohistochemistry score of 3+ and negative for a score of 0 or 1+, whereas cases with a score of 2+ underwent fluorescence *in situ* hybridization to determine gene amplification status; and ③ the cutoff value for the Ki-67cell proliferation index was 14%.

LVI was assessed in postoperative formalin-fixed, paraffin-embedded surgical specimens by two experienced pathologists, with results typically available within 2–3 days after surgery. Because LVI status is unavailable preoperatively, the nomogram was specifically designed for use in the early postoperative period, once pathological findings become available, to refine HNB risk estimates and guide adjuvant treatment.

### 2.4 Statistical analysis

All statistical analyses were conducted using the R programming environment (version 4.5.3). Continuous variables conforming to a normal distribution are presented as mean ± standard deviation (x̄ ± s), and between-group comparisons were performed using the independent samples t-test. For continuous variables that did not follow a normal distribution, data are expressed as median (interquartile range, IQR), and the Wilcoxon rank-sum test was applied for intergroup comparisons. Categorical variables are reported as frequencies (percentages), and between-group differences were assessed using the chi-square test or Fisher’s exact test, as appropriate.

Restricted cubic spline (RCS) functions were employed to examine potential non-linear associations between continuous variables (including age, maximum tumor diameter, and Ki-67 proliferation index) and HNB incidence. No statistically significant non-linear relationships were identified for any of these variables (all *P* > 0.05); therefore, they were incorporated into subsequent models in their linear form. All continuous variables (age and maximum tumor diameter) underwent Z-score standardization, which involved subtracting the population mean and dividing by the standard deviation. The beta coefficients and odds ratios (ORs) reported in subsequent logistic regression analyses were based exclusively on these standardized continuous variables, where an OR represented the multiplicative change in the risk of HNB occurrence per one standard deviation increase in the corresponding variable. Categorical variables were entered into the regression models as dummy variables.

The predictive model was developed using the following sequential steps:

Univariate logistic regression analysis: The independent association between each variable and HNB was evaluated separately. Variables with a *P*-value < 0.1 were retained as potential predictive factors for further analysis, serving as the pre-specified, initial screening threshold.

Multicollinearity assessment: The variance inflation factor (VIF) was calculated to detect multicollinearity among the variables selected during the initial screening. A VIF value < 2 was considered indicative of no clinically significant multicollinearity.

Multivariable logistic regression analysis: All variables that passed the initial screening were simultaneously entered into the multivariable logistic regression model. The forward stepwise selection method was applied to identify independent predictive factors, with a *P*-value of < 0.05 set as the threshold for statistical significance.

Receiver operating characteristic (ROC) curve analysis: ROC curves were plotted to evaluate the discriminative performance of the combined prediction model for HNB by calculating the area under the curve (AUC). Pairwise comparisons of the AUC values were performed using the DeLong test.

Nomogram construction: A visual nomogram model was developed by converting the regression coefficients of each independent predictive factor into an intuitive point-based scoring system based on the results of the final multivariable logistic regression analysis. The agreement between the model-predicted probabilities and the actual observed occurrence probabilities of HNB was assessed using calibration curves and the Hosmer-Lemeshow goodness-of-fit test. The clinical net benefit and practical utility of the model were evaluated using decision a curve analysis (DCA).

All statistical tests were two-sided, and a *P*-value of < 0.05 was considered statistically significant.

## 3 Results

### 3.1 Patient baseline characteristics

The proportions of patients with posterior echo attenuation, microcalcification, claw sign, angular margin sign, spiculation sign (significant long spicules/short spicules), suspicious axillary lymph node status, and lymphovascular invasion were all higher in the high-load group than in the low-load group (all P < 0.05, **Table 1**).

**Table 1:** Patient baseline characteristics.

Variable	Low burden (*n* = 84)	High burden (*n* = 91)	*t/χ*² value	*P*-value
Age (years)	52.29 ± 10.93	53.08 ± 9.80	0.504	0.615
Maximum tumor diameter (cm)	2.55 ± 1.30	2.81 ± 1.18	1.387	0.167
Lesion side	–	–	0.204	0.651
Right	49 (58.33)	50 (54.95)	–	–
Left	35 (41.67)	41 (45.05)	–	–
Quadrant	–	–	3.240	0.072
Upper outer quadrant	52 (61.90)	44 (48.35)	–	–
Other	32 (38.10)	47 (51.65)	–	–
Aspect ratio	–	–	0.021	0.886
<1	78 (92.86)	85 (93.41)	–	–
>1	6 (7.14)	6 (6.59)	–	–
Posterior echo	–	–	4.457	0.035
Attenuation	24 (28.57)	40 (43.96)	–	–
No change	60 (71.43)	51 (56.04)	–	–
Calcification	–	–	7.788	0.020
Coarse	4 (4.76)	1 (1.10)	–	–
Clustered	3 (3.57)	3 (3.30)	–	–
Microcalcification	45 (53.57)	66 (72.53)	–	–
None	32 (38.10)	21 (23.08)	–	–
Shape	–	–	2.536	0.111
Irregular	75 (89.29)	87 (95.60)	–	–
Sub-irregular	9 (10.71)	4 (4.40)	–	–
Crab claw sign	–	–	7.599	0.006
Absent	78 (92.86)	71 (78.02)	–	–
Present	6 (7.14)	20 (21.98)	–	–
Margin	–	–	0.143	0.931
Indistinct	44 (52.38)	50 (54.95)	–	–
Poorly defined	28 (33.33)	28 (30.77)	–	–
Relatively clear	12 (14.29)	13 (14.29)	–	–
Histological subtype	–	–	0.460	0.795
Invasive ductal carcinoma	72 (85.71)	81 (89.01)	–	–
Invasive lobular carcinoma	8 (9.52)	7 (7.69)	–	–
Other special subtypes	4 (4.76)	3 (3.30)	–	–
Angular margin	–	–	18.428	<0.001
Present	50 (59.52)	80 (87.91)	–	–
Absent	34 (40.48)	11 (12.09)	–	–
Spiculation sign	–	–	11.290	0.004
Marked long spicules	1 (1.19)	8 (8.79)	–	–
Few short spicules	6 (7.14)	17 (18.68)	–	–
No spicules	77 (91.67)	66 (72.53)	–	–
Echo type	–	–	0.818	0.366
Hypoechoic	81 (96.43)	85 (93.41)	–	–
Mixed echo	3 (3.57)	6 (6.59)	–	–
Echo homogeneity	–	–	1.719	0.190
Heterogeneous	21 (25.00)	31 (34.07)	–	–
Homogeneous	63 (75.00)	60 (65.93)	–	–
Blood flow pattern	–	–	4.726	0.317
Penetrating	9 (10.71)	12 (13.19)	–	–
Punctate	20 (23.81)	24 (26.37)	–	–
Branched	8 (9.52)	10 (10.99)	–	–
No blood flow	15 (17.86)	8 (8.79)	–	–
Linear	32 (38.10)	37 (40.66)	–	–
Lymph node status	–	–	15.576	<0.001
Suspicious	51 (60.71)	79 (86.81)	–	–
Normal	33 (39.29)	12 (13.19)	–	–
Histological grade	–	–	1.041	0.595
Grade 1	3 (3.57)	4 (4.40)	–	–
Grade 2	48 (57.14)	45 (49.45)	–	–
Grade 3	33 (39.29)	42 (46.15)	–	–
Estrogen receptor (ER)	–	–	3.549	0.060
Positive	58 (69.05)	74 (81.32)	–	–
Negative	26 (30.95)	17 (18.68)	–	–
Progesterone receptor (PR)	–	–	1.387	0.239
Positive	52 (61.90)	64 (70.33)	–	–
Negative	32 (38.10)	27 (29.67)	–	–
Human epidermal growth factor receptor 2 (HER-2)	–	–	0.284	0.594
Positive	21 (25.00)	26 (28.57)	–	–
Negative	63 (75.00)	65 (71.43)	–	–
Ki-67 proliferation index	–	–	2.244	0.134
Low expression	32 (38.10)	25 (27.47)	–	–
High expression	52 (61.90)	66 (72.53)	–	–
Lymphovascular invasion	–	–	8.055	0.005
Absent	54 (64.29)	39 (42.86)	–	–
Present	30 (35.71)	52 (57.14)	–	–

### 3.2 Univariate logistic regression analysis

The single-factor analysis revealed that eight variables/variable groups, namely quadrant (other), posterior echo (no change), claw sign (present), angular margin sign (absent), spiculation sign (absent), lymph node status (normal), ER (negative), and lymphovascular invasion (present), were statistically associated with HNB (all *P* < 0.1). The OR values, confidence intervals, and Wald test P-values for each variable at specific levels are shown in **Table 2**.

**Table 2:** Univariate logistic regression analysis.

Variable	Level	*β*	*SE*	*Wald*	*P*	*OR*	95% *CI* lower limit	95% *CI* upper limit
Age	Continuous (per 1 SD)	0.077	0.152	0.257	0.612	1.080	0.802	1.456
Maximum tumor diameter (cm)	Continuous (per 1 SD)	0.223	0.161	1.922	0.166	1.250	0.921	1.714
Lesion side	Right (Reference)	–	–	–	–	1.000	–	–
Lesion side	Left	0.138	0.306	0.204	0.651	1.148	0.631	2.089
Quadrant	Upper outer quadrant (Reference)	–	–	–	–	1.000	–	–
Quadrant	Other	0.551	0.307	3.219	0.073	1.736	0.950	3.171
Aspect ratio	<1 (Reference)	–	–	–	–	1.000	–	–
Aspect ratio	>1	-0.086	0.598	0.021	0.886	0.918	0.284	2.964
Posterior echo	Attenuation (Reference)	–	–	–	–	1.000	–	–
Posterior echo	No change	-0.673	0.321	4.404	0.036	0.510	0.272	0.956
Calcification	Coarse (Reference)	–	–	–	–	1.000	–	–
Calcification	Clustered	1.386	1.384	1.003	0.317	4.000	0.265	60.325
Calcification	Microcalcification	1.769	1.135	2.432	0.119	5.867	0.635	54.223
Calcification	None	0.965	1.153	0.701	0.402	2.625	0.274	25.140
Shape	Irregular (Reference)	–	–	–	–	1.000	–	–
Shape	Sub-irregular	-0.959	0.621	2.385	0.123	0.383	0.113	1.295
Crab claw sign	Absent (Reference)	–	–	–	–	1.000	–	–
Crab claw sign	Present	1.298	0.494	6.917	0.009	3.662	1.392	9.634
Margin	Indistinct (Reference)	–	–	–	–	1.000	–	–
Margin	Poorly defined	-0.128	0.338	0.143	0.705	0.880	0.454	1.706
Margin	Relatively clear	-0.048	0.451	0.011	0.916	0.954	0.395	2.305
Angular margin	Present (Reference)	–	–	–	–	1.000	–	–
Angular margin	Absent	-1.598	0.391	16.720	<0.001	0.202	0.094	0.435
Spiculation sign	Marked long spicules (Reference)	–	–	–	–	1.000	–	–
Spiculation sign	Few short spicules	-1.038	1.162	0.798	0.372	0.354	0.036	3.455
Spiculation sign	No spicules	-2.234	1.074	4.326	0.038	0.107	0.013	0.879
Echo type	Hypoechoic (Reference)	–	–	–	–	1.000	–	–
Echo type	Mixed echo	0.645	0.724	0.794	0.373	1.906	0.461	7.876
Echo homogeneity	Heterogeneous (Reference)	–	–	–	–	1.000	–	–
Echo homogeneity	Homogeneous	0.438	0.335	1.709	0.191	1.550	0.803	2.990
Blood flow pattern	Penetrating (Reference)	–	–	–	–	1.000	–	–
Blood flow pattern	Punctate	-0.105	0.535	0.039	0.844	0.900	0.315	2.568
Blood flow pattern	Branched	-0.065	0.648	0.010	0.921	0.938	0.263	3.336
Blood flow pattern	No blood flow	-0.916	0.621	2.174	0.140	0.400	0.118	1.328
Blood flow pattern	Linear	-0.143	0.503	0.080	0.777	0.867	0.324	2.323
Lymph node status	Suspicious (Reference)	–	–	–	–	1.000	–	–
Lymph node status	Normal	-1.449	0.382	14.395	<0.001	0.235	0.111	0.497
Histological grade	Grade 1 (Reference)	–	–	–	–	1.000	–	–
Histological grade	Grade 2	-0.352	0.791	0.198	0.656	0.703	0.149	3.317
Histological grade	Grade 3	-0.047	0.798	0.003	0.954	0.956	0.200	4.565
Estrogen receptor (ER)	Positive (Reference)	–	–	–	–	1.000	–	–
Estrogen receptor (ER)	Negative	-0.669	0.358	3.490	0.062	0.512	0.254	1.033
Progesterone receptor (PR)	Positive (Reference)	–	–	–	–	1.000	–	–
Progesterone receptor (PR)	Negative	-0.378	0.321	1.382	0.240	0.686	0.365	1.286
Human epidermal growth factor receptor 2 (HER-2)	Positive (Reference)	–	–	–	–	1.000	–	–
Human epidermal growth factor receptor 2 (HER-2)	Negative	-0.182	0.343	0.283	0.595	0.833	0.426	1.631
Ki-67 proliferation index	Low expression (Reference)	–	–	–	–	1.000	–	–
Ki-67 proliferation index	High expression	0.485	0.325	2.229	0.135	1.625	0.859	3.072
Lymphovascular invasion	Absent (Reference)	–	–	–	–	1.000	–	–
Lymphovascular invasion	Present	0.875	0.311	7.924	0.005	2.400	1.305	4.415

### 3.3 Multivariate logistic regression analysis

All eight variables that met the initial screening criteria (*P* < 0.1) in the single-factor analysis were included in the multivariate logistic regression analysis. The multicollinearity test showed that the VIF values of all variables were < 1.5, indicating that there was no significant multicollinearity problem. The results showed that the absence of the marginal angular margin sign and normal axillary lymph node status were independent protective factors for HNB, while the presence of lymphovascular invasion was an independent risk factor (all *P* < 0.05); the other variables did not reach statistical significance. Details are shown in **Table 3**.

**Table 3:** Multivariate logistic regression analysis.

Variable	*β*	*SE*	*Wald*	*OR*	95% *CI* lower limit	95% *CI* upper limit	*P*
Absent angular margin	-1.660	0.418	15.745	0.190	0.084	0.432	<0.001
Normal axillary lymph node status	-1.573	0.415	14.383	0.208	0.089	0.456	<0.001
Present lymphovascular invasion	0.996	0.350	8.093	2.707	1.364	5.377	0.004

Only statistically significant variables are listed; Intercept *OR* = 1.513 (95%*CI*: 0.917–2.533, *P* = 0.109).

### 3.4 The predictive value of relevant factors for HNB

The regression coefficients of the independent influencing factors (angular margin sign, lymph node status, and lymphovascular invasion) in the multivariate logistic regression analysis were combined with the constant term to calculate the predicted probability for each patient. This probability was used as a combined predictive factor in the ROC analysis. The results showed that the combination of the angular margin sign, lymph node status, and lymphovascular invasion had a higher AUC value for HNB prediction than individual detection (*Z* = 4.176, 3.781, and 3.672, respectively, *P* < 0.05; **Table 4**; **Figure 2**).

**Table 4:** The predictive value of relevant factors for HNB.

Item	AUC	Standard error	95% *CI*	*P*	Youden index	Cut-off value	Sensitivity (%)	Specificity (%)
Angular margin	0.642	0.032	0.566~0.713	<0.001	0.284	–	87.91	40.48
Lymph node status	0.630	0.032	0.554~0.702	<0.001	0.261	–	86.81	39.29
Lymphovascular invasion	0.607	0.037	0.531~0.680	<0.001	0.214	–	57.14	64.29
Combined model	0.758*	0.035	0.687~0.819	<0.001	0.383	0.438	83.52	54.76

* indicates *P* < 0.05 compared with single detection.

**Figure 2:**
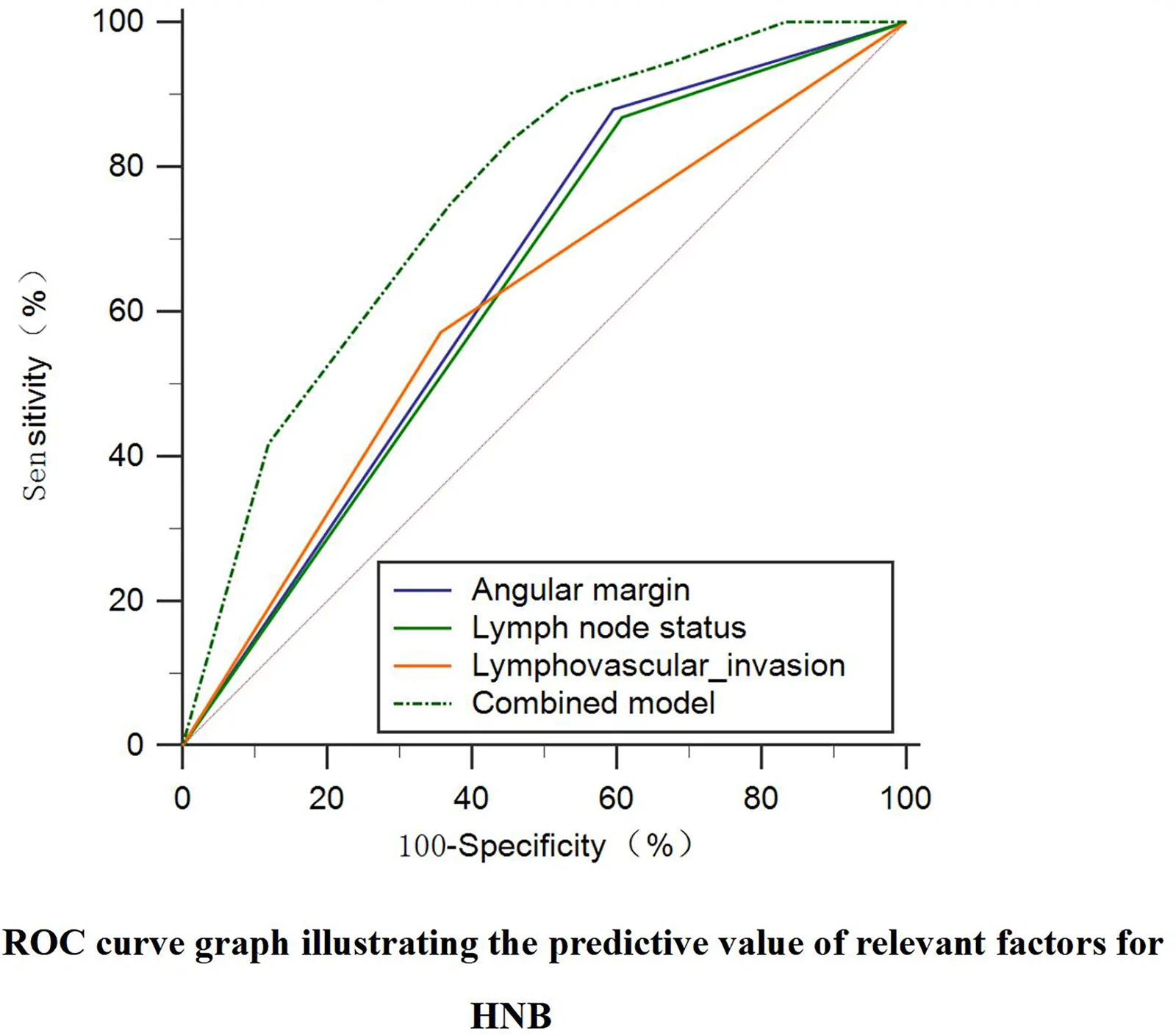
ROC curve graph illustrating the predictive value of relevant factors for HNB.

### 3.5 Comparison of baseline characteristics between the model and validation groups

There was no statistically significant difference in the baseline characteristics between the model and validation groups (*P* > 0.05), indicating that the model and validation groups were highly consistent (**Table 5**).

**Table 5:** Comparison of baseline characteristics between the model and validation groups.

Variable	Model group (*n* = 175)	Validation group (*n* = 86)	*t/χ*² value	*P*-value
Age (years)	52.69 ± 10.35	51.74 ± 9.87	0.708	0.480
Maximum tumor diameter (cm)	2.68 ± 1.24	2.75 ± 1.32	0.420	0.675
Lesion side	–	–	0.919	0.338
Right	99 (56.57)	54 (62.79)	–	–
Left	76 (43.43)	32 (37.21)	–	–
Quadrant	–	–	1.079	0.299
Upper outer quadrant	96 (54.86)	53 (61.63)	–	–
Other	79 (45.14)	33 (38.37)	–	–
Aspect ratio	–	–	0.140	0.708
<1	163 (93.14)	79 (91.86)	–	–
>1	12 (6.86)	7 (8.14)	–	–
Posterior echo	–	–	3.152	0.076
Attenuation	64 (36.57)	22 (25.58)	–	–
No change	111 (63.43)	64 (74.42)	–	–
Calcification	–	–	4.264	0.119
Coarse	5 (2.86)	6 (6.98)	–	–
Clustered	6 (3.43)	5 (5.81)	–	–
Microcalcification	111 (63.43)	46 (53.49)	–	–
None	53 (30.29)	29 (33.72)	–	–
Shape	–	–	3.780	0.051
Irregular	162 (92.57)	73 (84.89)	–	–
Sub-irregular	13 (7.43)	13 (15.12)	–	–
Crab claw sign	–	–	0.038	0.846
Absent	149 (85.14)	74 (86.05)	–	–
Present	26 (14.86)	12 (13.95)	–	–
Margin	–	–	2.034	0.362
Indistinct	94 (53.71)	50 (58.14)	–	–
Poorly defined	56 (32.00)	29 (33.72)	–	–
Relatively clear	25 (14.29)	7 (8.14)	–	–
Angular margin	–	–	1.627	0.202
Present	130 (74.29)	70 (81.40)	–	–
Absent	45 (25.71)	16 (18.60)	–	–
Spiculation sign	–	–	2.520	0.284
Marked long spicules	9 (5.14)	1 (1.16)	–	–
Few short spicules	23 (13.14)	11 (12.79)	–	–
No spicules	143 (81.71)	74 (86.05)	–	–
Echo type	–	–	0.360	0.549
Hypoechoic	166 (94.86)	83 (96.51)	–	–
Mixed echo	9 (5.14)	3 (3.49)	–	–
Echo homogeneity	–	–	1.691	0.193
Heterogeneous	52 (29.71)	19 (22.09)	–	–
Homogeneous	123 (70.29)	67 (77.91)	–	–
Blood flow pattern	–	–	0.467	0.976
Penetrating	21 (12.00)	9 (10.47)	–	–
Punctate	44 (25.14)	22 (25.58)	–	–
Branched	18 (10.29)	8 (9.30)	–	–
No blood flow	23 (13.14)	10 (11.63)	–	–
Linear	69 (39.43)	37 (43.02)	–	–
Lymph node status	–	–	1.820	0.177
Suspicious	130 (74.29)	57 (66.28)	–	–
Normal	45 (25.71)	29 (33.72)	–	–
Histological grade	–	–	0.456	0.796
Grade 1	7 (4.00)	5 (5.81)	–	–
Grade 2	93 (53.14)	44 (51.16)	–	–
Grade 3	75 (42.86)	37 (43.02)	–	–
Estrogen receptor (ER)	–	–	1.173	0.279
Positive	132 (75.43)	70 (81.40)	–	–
Negative	43 (24.57)	16 (18.60)	–	–
Progesterone receptor (PR)	–	–	0.897	0.344
Positive	116 (66.29)	62 (72.09)	–	–
Negative	59 (33.71)	24 (27.91)	–	–
Human epidermal growth factor receptor 2 (HER-2)	–	–	0.392	0.531
Positive	47 (26.86)	20 (23.26)	–	–
Negative	128 (73.14)	66 (76.74)	–	–
Ki-67 proliferation index	–	–	0.586	0.444
Low expression	57 (32.57)	24 (27.91)	–	–
High expression	118 (67.43)	62 (72.09)	–	–
Lymphovascular invasion	–	–	0.091	0.763
Absent	93 (53.14)	44 (51.16)	–	–
Present	82 (46.86)	42 (48.84)	–	–

### 3.6 Construction of a nomogram

A nomogram for predicting HNB risk was developed using logistic regression. It incorporates three predictors: marginal angular margin sign, lymph node status, and lymphovascular invasion. Of these, the marginal angular margin sign carried the greatest weight in the model, highlighting its important contribution to risk estimation (**Figure 3**). The model achieved an AUC of 0.758 (95% CI: 0.689–0.819), and the validation cohort showed a similar AUC of 0.782 (95% CI: 0.684–0.880) (**Figure 4**). The calibration plot demonstrated good agreement between the predicted probabilities and observed outcomes (**Figure 5**). In DCA, the nomogram provided a net clinical benefit when applied across a threshold probability range of 0.1-0.6 (**Figure 6**).

**Figure 3:**
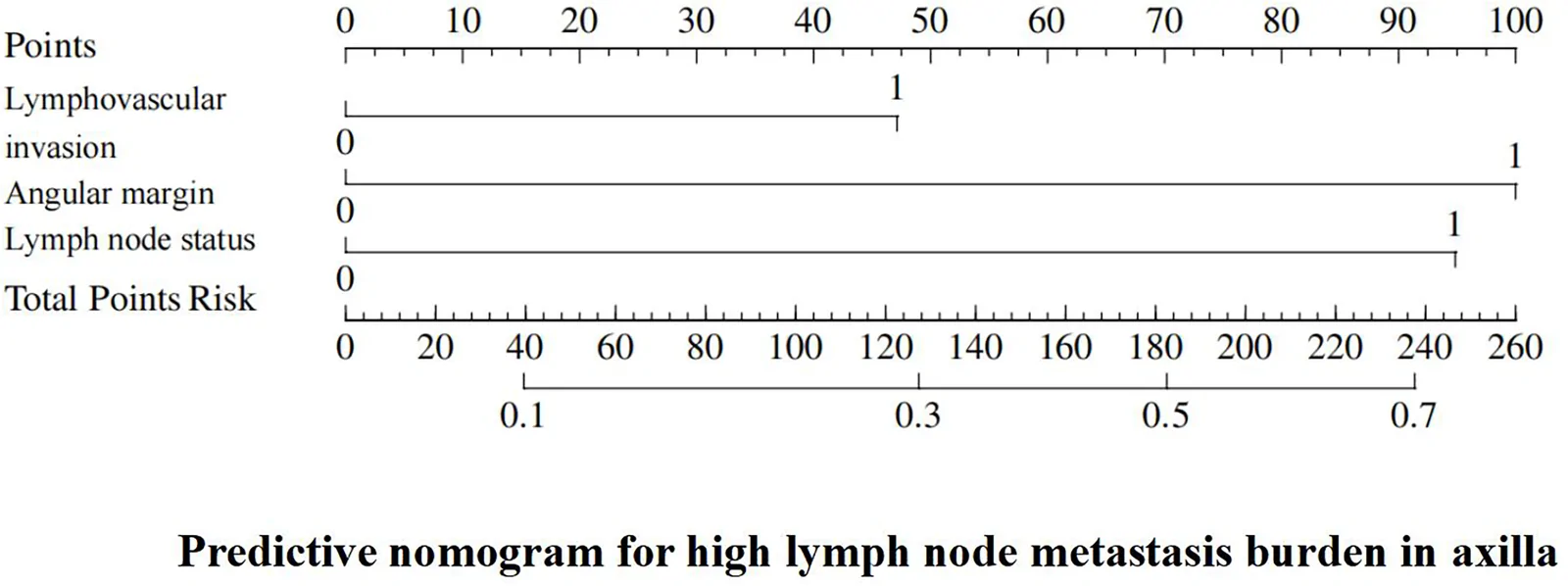
Predictive nomogram for high lymph node metastasis burden in axilla.

**Figure 4:**
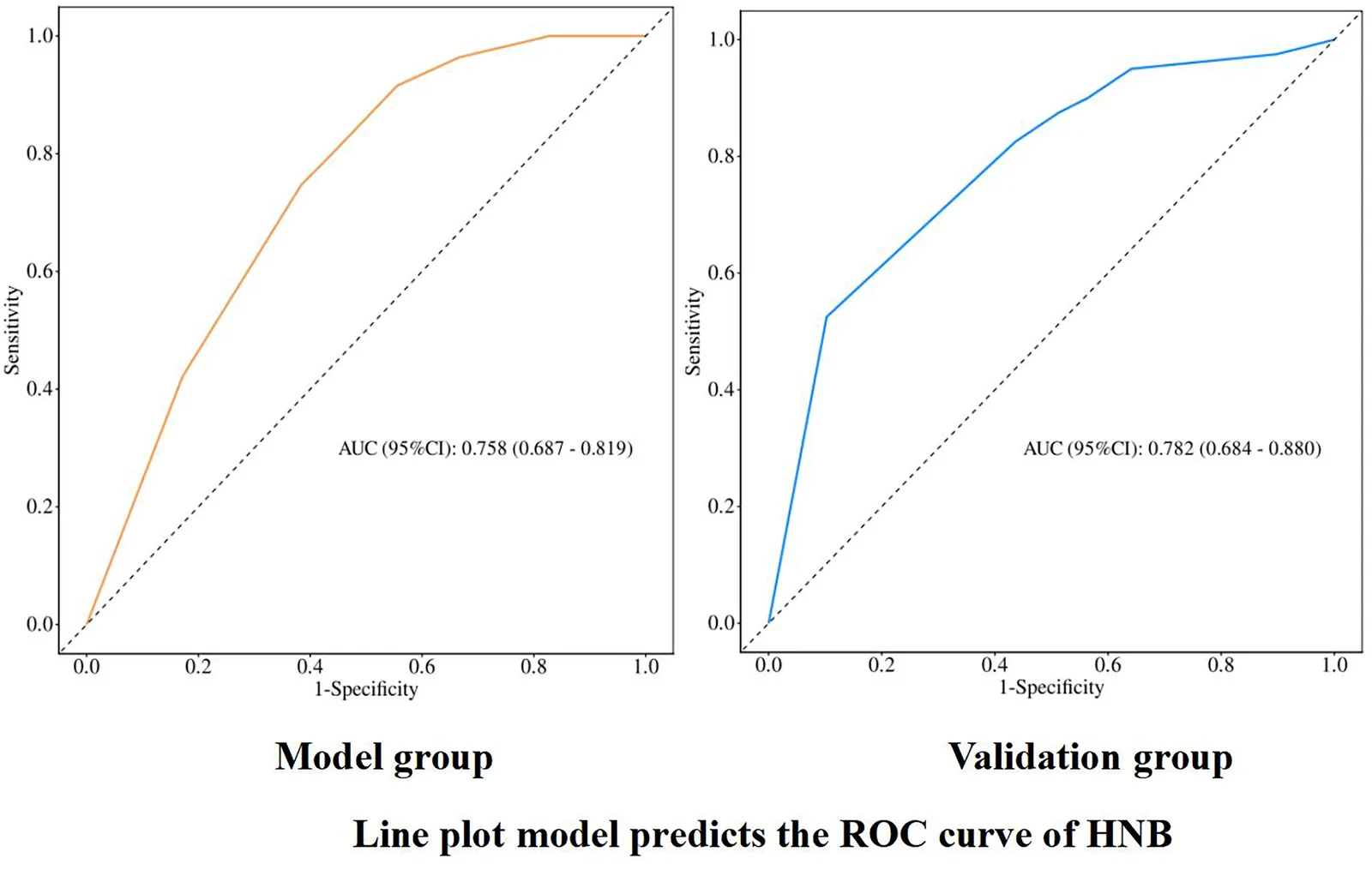
Line plot model predicts the ROC curve of HNB.

**Figure 5:**
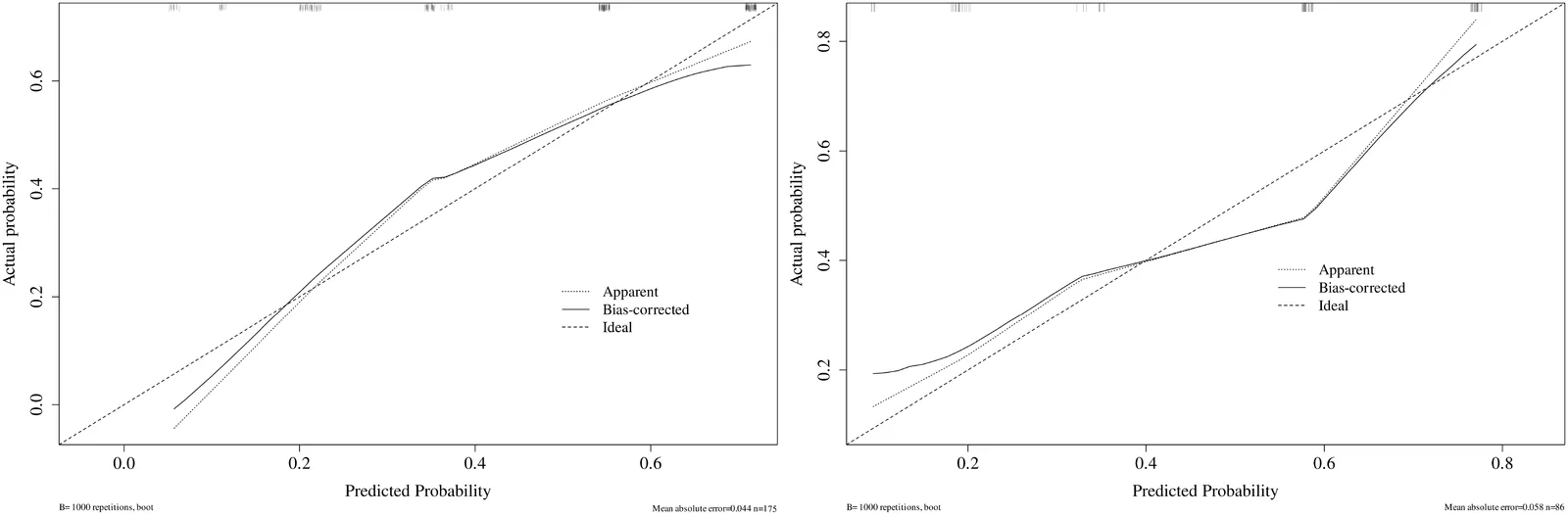
Bootstrap calibration curve.

**Figure 6:**
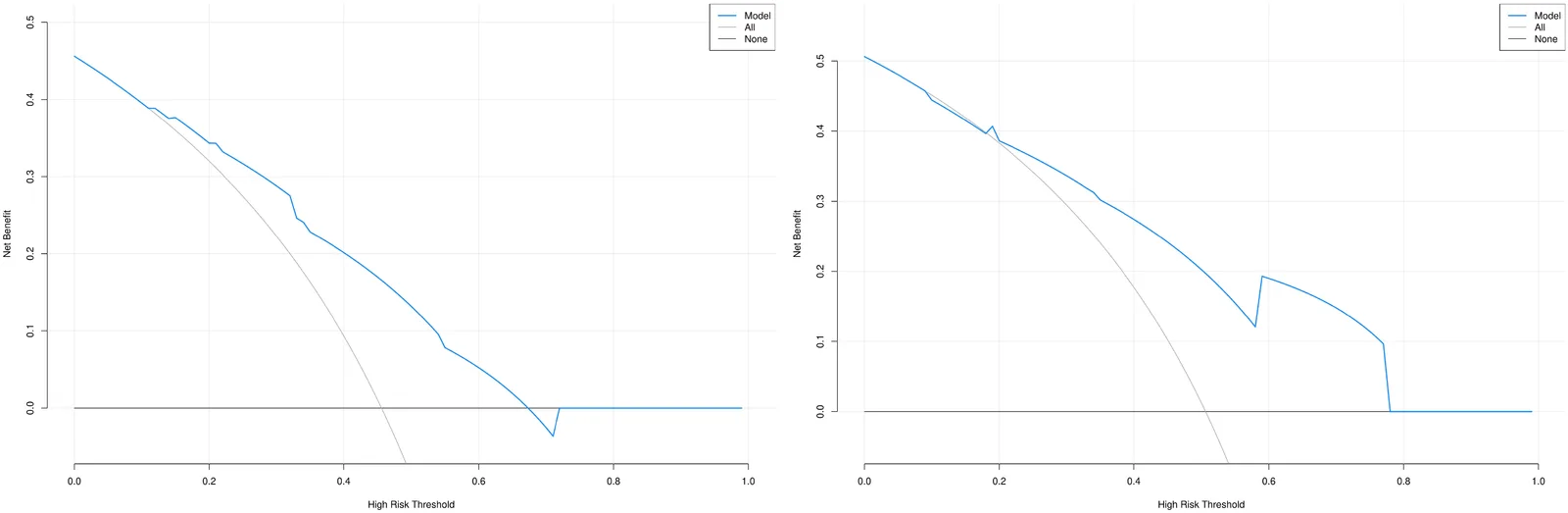
Decision curve analysis.

## 4 Discussion

The axillary lymph node metastatic burden is a key factor influencing the prognosis and treatment decisions in patients with invasive breast cancer. Accurate identification of patients with HNB is essential for guiding adjuvant treatment strategies, including determining the extent of radiotherapy fields, the need for completion axillary lymph node dissection when SLNB is initially performed, and the intensity of systemic therapy. However, risk stratification in the early postoperative period remains suboptimal. In this retrospective study of 175 patients with node-positive invasive breast cancer, three independent predictors of HNB were identified and incorporated into a simple nomogram. The nomogram is intended for use during the early postoperative period, when both ultrasound findings and pathological LVI results are available to facilitate timely and individualized adjuvant treatment planning.

The results of the multivariate analysis in this study showed that the angular margin sign of the primary lesion was the strongest independent predictor of HNB (*OR* = 0.190), and patients without the angular margin sign had an 81% lower risk of developing HNB. The angular margin sign is a typical ultrasound sign of invasive tumor growth, and its formation mechanism is closely related to the high expression of matrix metalloproteinases (MMPs) in tumor cells. MMPs can degrade the extracellular matrix and basement membranes, promoting the invasive growth of tumor cells toward surrounding tissues and creating conditions for tumor cells to invade lymphatic vessels and undergo distant metastasis. This study confirmed that the angular margin sign of the primary breast cancer lesion is closely related to axillary lymph node metastasis (15). This study also found that it was significantly associated with the metastatic burden, suggesting that the higher the degree of invasive tumor growth, the greater the risk of multiple lymph node. Ultrasound status of axillary lymph nodes was another important independent protective factor for HNB (*OR* = 0.208). Patients with normal ultrasound indications of lymph nodes had a 79.2% lower risk of developing HNB. Conventional ultrasound can effectively identify metastatic lymph nodes by observing their morphology, cortical thickness, lymphoid gate structure, and blood supply pattern of the lymph nodes. In this study, the proportion of axillary lymph nodes with suspicious ultrasound findings in the HNB group was as high as 86.8%, significantly higher than the 60.7% in the low-load group, which is consistent with the results of previous studies (16, 17). However, ultrasound has limited sensitivity in identifying micrometastases, and approximately 10% to 15% of patients with negative ultrasound results may still have lymph node metastasis. Therefore, the possibility of HNB cannot be completely ruled out based solely on ultrasound results. Lymphovascular invasion was the only independent clinical pathological risk factor in this study (*OR* = 2.707). The risk of HNB in patients with positive lymphovascular invasion was 2.7 times that of patients with negative lymphovascular invasion. Lymphovascular invasion is a key step in tumor metastasis. When tumor cells invade blood vessels or lymphatic vessels, they can return to regional lymph nodes or distant organs via lymph fluid or blood, forming metastatic foci in these sites. Studies have shown (13) a direct relationship between lymphovascular invasion and the number of involved axillary lymph nodes in patients with breast cancer. Patients with positive lymphovascular invasion are more likely to develop multiple lymph node metastases. The results of this study further support this conclusion.

Some relevant variables in traditional cognition did not enter the final model. Although the maximum diameter of the tumor showed a certain correlation trend in the univariate analysis (*P* = 0.167), it did not reach the screening threshold (*P* < 0.1) and was not included in the subsequent analysis. The conclusions of previous studies regarding whether tumor size is an independent predictor of HNB remain unclear. Some studies have confirmed it as an independent risk factor in multivariate analysis (18), while other studies have found that after introducing ultrasound or imaging features, the independent predictive value of tumor size weakened or even disappeared (19, 20).This study is in line with the aforementioned viewpoint, suggesting that ultrasonic characteristics reflecting the biological behavior of tumors may have greater predictive advantages than simple anatomical dimensions. Ki-67, a proliferation activity marker, did not reach the initial screening standard in this study (*P* = 0.135) and was thus not included in the subsequent analysis. Its association with HNB also remains controversial in the literature. Some studies support its independent value, while others (6, 18) found that although Ki-67 was significant in the univariate analysis in a model with a high lymph node burden (≥ 3 nodes) as the outcome, it did not become an independent predictor in the multivariate analysis. Similar findings were observed in this study, indicating that the independent value of Ki-67 in the preoperative non-invasive prediction of HNB may be limited and that its predictive information may have been covered by other variables, such as ultrasonic characteristics.

In terms of model performance, the AUC of the logistic regression model in this study was 0.758. Previous logistic regression models based on radiomics, conventional ultrasound, and automatic breast volumetric ultrasound (ABVS) imaging biomarkers achieved AUC values of 0.846, 0.802, and 0.827 in the training group, respectively, and the AUC values in the validation group ranged from 0.733 to 0.768 (21–23). This study showed a similar performance to the above-mentioned model validation group, suggesting that our model is highly robust. Although the AUC of this model was slightly lower than that of some multivariate or radiomics models, the nomogram model constructed in this study has the following advantages: (1) Simple and practical indicators: only three clinical routine examination indicators were included, no additional laboratory tests or complex image post-processing were required, and it is easy to be applied in primary hospitals; (2) Good predictive performance: the model’s AUC was 0.758, and the validation group’s AUC was 0.782, which was comparable to the performance of most models that included more indicators (21–23); and (3) Strong clinical applicability: On decision curve analysis, the model offered substantial net clinical benefit across a broad span of risk thresholds.

The proposed nomogram is designed for use shortly after surgery, when the pathological report, including LVI status, becomes available (typically within 2–3 days postoperatively); however, the definitive axillary nodal burden may remain unknown in patients who initially underwent SLNB. At this stage, several important treatment decisions must be considered.

1. Completion ALND: If SLNB is initially performed and the predicted probability of HNB is high, completion ALND may be considered to improve regional disease control.
2. Radiotherapy planning: Patients at high risk of HNB may require extended-field radiotherapy, including irradiation of the supraclavicular or internal mammary lymph nodes. The nomogram may help radiation oncologists identify patients who could benefit from comprehensive nodal irradiation.
3. Systemic therapy intensification: Because HNB is associated with an increased risk of distant recurrence, the estimated probability generated by the nomogram may help inform decisions regarding chemotherapy intensification, the addition of bisphosphonates, and more intensive endocrine therapy.

By providing a quantitative risk estimate within days after surgery, this nomogram may help bridge the gap between pathological assessment and adjuvant treatment planning, thereby facilitating timely and individualized care.

This study has several limitations. First, the retrospective, single-center design may have introduced a selection bias. Second, the sample size was limited, and although temporal internal validation was performed, external validation in other institutions is required to confirm the model generalizability. Third, LVI, a predictor included in the nomogram, was assessed using postoperative pathological specimens. Therefore, the model is intended for early postoperative use rather than preoperative use. Although this limits its preoperative applicability, it is consistent with the model’s intended purpose of guiding adjuvant treatment decisions once the pathological results become available. Fourth, breast MRI findings, inflammatory markers, and radiomic features were not included, although they may provide additional information. Nevertheless, the use of conventional ultrasound features and routine pathological indicators enhances the model’s accessibility and feasibility for daily clinical practice. Fifth, subtype-specific comparative analyses based on histopathological subtypes (e.g., invasive ductal carcinoma vs. invasive lobular carcinoma) were not performed in this study. Given that radiological presentations and metastatic biological behaviors often differ significantly across pathological subtypes of invasive breast cancer, the predictive performance and generalizability of the nomogram for rare special subtypes still need to be further verified in larger-scale cohorts. Finally, molecular subtype analyses were not performed because of the small sample size.

## 5 Conclusion

We developed and internally validated a nomogram incorporating preoperative ultrasound features (angular margin sign and axillary lymph node status) and postoperative LVI to predict HNB in patients with node-positive invasive breast cancer. Designed specifically for early postoperative use, the model demonstrated good discrimination, calibration, and clinical utility in our study. By providing a quantitative risk estimate within days after surgery, it may assist multidisciplinary teams in making timely and individualized adjuvant treatment decisions, including those regarding completion ALND, radiotherapy field design, and systemic therapy intensification. Prospective external validation is warranted to confirm the generalizability of the model before its widespread clinical implementation.

## Data Availability

The original contributions presented in the study are included in the article/supplementary material. Further inquiries can be directed to the corresponding author.
